# Wastewater-based surveillance for severe acute respiratory syndrome coronavirus 2 variants in Gwangju, Republic of Korea

**DOI:** 10.3389/fmicb.2025.1676742

**Published:** 2025-09-22

**Authors:** Chaeyoung Lee, Jungwook Park, Jin Sun No, Jongpil Kim, Jungmi Seo, Dongju Kim, Il-Hwan Kim, Eun-Jin Kim

**Affiliations:** ^1^Division of Emerging Infectious Diseases, Department of Laboratory Diagnosis and Analysis, Korea Disease Control and Prevention Agency, Cheongju-si, Republic of Korea; ^2^Division of Emerging Infectious Disease, Department of Infectious Disease Research, Health and Environment Research Institute of Gwangju, Gwangju, Republic of Korea

**Keywords:** COVID-19, SARS-CoV-2, wastewater-based surveillance, genomic surveillance, Republic of Korea

## Abstract

The coronavirus disease pandemic has underscored the importance of surveillance systems for timely response to public health threats. Genomic surveillance of severe acute respiratory syndrome coronavirus 2 (SARS-CoV-2) is essential for tracking the emergence and dissemination of variants of the virus. However, as clinical surveillance is conducted primarily among symptomatic individuals and their contacts, the ability to detect asymptomatic infections and undiagnosed cases is limited. Accordingly, in this study, we evaluated the utility of wastewater-based surveillance as a complementary approach to traditional clinical monitoring methods. From epidemiological weeks 1–21 in 2024, samples were collected from three wastewater treatment plants in the Gwangju region of South Korea. Quantification of SARS-CoV-2 RNA and whole-genome sequencing revealed that temporal changes in the viral concentration closely mirrored the confirmed case counts. Comparative analysis was conducted of the Illumina MiSeq and NextSeq platforms, revealing that the NextSeq platform exhibited relatively higher sensitivity in sequencing depth and genome coverage. Analysis of wastewater-derived sequences further revealed that the sublineage diversity in the samples closely resembled that of contemporaneous clinical isolates. Our findings highlight the utility of wastewater-based surveillance as an effective adjunct to conventional systems, as the method enhances the capacity to monitor SARS-CoV-2 transmission dynamics and variant emergence within the community.

## 1 Introduction

The coronavirus disease (COVID-19) pandemic has highlighted the critical role of surveillance systems in guiding public health interventions (Morgan et al., 2021). In particular, genomic surveillance through the sequencing of viral genomes from individuals infected with the severe acute respiratory syndrome coronavirus 2 (SARS-CoV-2) has been pivotal in identifying emerging variants and tracking their transmission dynamics (Kwon et al., 2021; Park et al., 2021; Park et al., 2022). Genomic surveillance based on clinical specimens primarily relies on the diagnostic testing of symptomatic individuals or their known contacts. However, this practice limits the detection of asymptomatic infections and undiagnosed cases.

In contrast, wastewater-based surveillance facilitates the detection of diverse pathogens in wastewater samples, provides a non-invasive means to forecast outbreaks, and acts as an early warning system of emerging novel variants. During the COVID-19 pandemic, numerous countries adopted this approach to complement clinical surveillance that is limited mainly to symptomatic individuals (Wu et al., 2022). Notably, in early 2020, Australia demonstrated that SARS-CoV-2 RNA concentrations in samples from six wastewater treatment plants (WWTPs) closely tracked the trajectory of reported COVID-19 cases. This finding validated the applicability of wastewater-based surveillance for public health response strategies (Ahmed et al., 2020).

In 2022, wastewater-based surveillance in Austria revealed a marked increase in viral genetic diversity during the infection wave of the Delta variant, offering a means to predict the emergence of novel variants (Amman et al., 2022). Similarly, an analytical framework implemented in Switzerland facilitated early detection of emerging SARS-CoV-2 lineages in wastewater samples, further substantiating the utility of wastewater-based genomic surveillance for variant monitoring (Jahn et al., 2022).

Since 2023, the Korea Disease Control and Prevention Agency (KDCA) has led a nationwide wastewater-based surveillance initiative, the Korea Wastewater Surveillance (KOWAS). This surveillance is done in collaboration with 17 regional health and environmental research institutes. KOWAS aims to characterize the regional patterns of infectious disease detection by quantifying the viral loads in wastewater samples (Jang et al., 2023). Although this approach is effective in tracking epidemiological trends based on viral concentrations, its ability to support variant tracking or phylogenetic analysis is limited.

Consequently, the need to incorporate whole-genome sequencing (WGS) into wastewater-based surveillance frameworks for clinical surveillance has been growing. In August 2023, Korea reclassified COVID-19 cases and shifted surveillance from mandatory to sentinel (Korea Disease Control and Prevention Agency, 2023). This shift significantly reduced the volume and scope of collected positive clinical specimens, causing a marked decrease in the number of samples available for genomic surveillance. The resulting surveillance gap raises concerns about delayed detection and tracking of emerging variants. Accordingly, establishing a complementary wastewater-based genomic surveillance system is essential to address the limitations of clinical specimen collection and ensure continuous monitoring of circulating variants at the community level.

In this study, wastewater samples were collected from three treatment plants in Gwangju between epidemiological weeks 1 and 21 in 2024. The SARS-CoV-2 concentrations were quantified and WGS was conducted using the Illumina MiSeq and NextSeq platforms (Illumina, Inc., California, USA). We compared the sequencing performance across platforms and analyzed contemporaneous clinical specimens, facilitating the assessment of the utility of wastewater-based surveillance for variant detection and its potential integration into the national public health monitoring systems.

## 2 Materials and methods

### 2.1 Wastewater sample collection

Influent wastewater samples were collected from three WWTPs in Gwangju between weeks 1 and 21 of 2024. One liter of wastewater influent was collected every week with the help of the Gwangju Environment Corporation by floating the influent directly from the WWTPs. The three WWTPs have a common treatment capacity of more than 10,000 tonne/day (Table 1). These WWTPs treat the wastewater of almost 100% of the total population of Gwangju. The A WWTP treats wastewater in four of the five districts of Gwangju, accounting for 83% of its population. The B WWTP treats wastewater from one of the five districts of Gwangju, accounting for 16% of the total population. The C WWTP treats wastewater in some densely populated areas of one of the districts of Gwangju, accounting for 1% of its total population (Republic of Korea e-government, 2024).

**TABLE 1:** Characteristics of WWTP.

	WWTPs		
Characteristics	A	B	C
Treatment capacity (m^3^/day)	600,000	120,000	16,000
Population	1,160,000	190,000	50,000
Sampling method	Direct sampling	Direct sampling	Direct sampling

### 2.2 Nucleic acid extraction and TaqMan array card for SARS-Cov2 (E and ORF1ab gene) detection

Ten milliliter of the wastewater influent was transferred into a 50 mL conical centrifuge tube. The samples were centrifuged for 30 min at 3000 × *g* (Centrifuge 5810R; Eppendorf, Germany) and the sample supernatant was used to extract SARS-CoV-2 nucleic acid by an automated machine (Chemagic 360 Nucleic Acid Extractor, PerkinElmer Inc., Massachusetts, USA). The reagent used in the equipment was a chemical viral DNA/RNA 10 K kit (#CMG-749, PerkinElmer Inc., Massachusetts, USA), and the nucleic acids were extracted according to the manufacturer’s protocol. For detecting SARS-CoV-2 (E and ORF1ab genes), we used a custom TaqMan Array Card (TAC) (Applied Biosystems, ThermoFischer Scientific, Massachusetts, USA), including the SARS-CoV-2 E gene, ORF gene primer-probe set. Before loading the card, a pre-amplifying process was performed on a Proflex polymerase chain reaction (PCR) thermal cycler instrument (Applied Biosystems, ThermoFischer Scientific, Massachusetts, USA). The mixture we used comprised Tacpath 1-step mastermix 2.5 uL, preamp primer for SARS-CoV-2 E gene, ORF1ab gene 2.5 uL, and sample nucleic acid 5 uL. The cycling conditions included incubation at 25 °C for 2 min, reverse transcription at 50 °C for 30 min, activation at 95 °C for 2 min, 14 cycles of denaturation at 95 °C for 15 s, annealing/extension at 60 °C for 2 min.

The card was loaded with 100 uL of reaction mix per port, containing pre-amplified product 10 uL, TaqMan Fast (ThermoFischer Scientific) advanced master mix 50 uL, and nuclease-free water 40 uL. The loaded card was centrifuged for 1 min at 1000 × *g* (Centrifuge 5810R, Eppendorf, Germany), sealed according to the manufacturer’s protocol and run on an Applied Biosystems Quantstudio 12 K Flex PCR System (ThermoFischer, Massachusetts, USA). The cycling conditions included activation at 95 °C for 10 min, amplification for 40 cycles at 95 °C for 3 s, followed by annealing/extension for 40 cycles at 95 °C for 30 s.

### 2.3 Whole-genome sequencing of wastewater

To obtain the genomic sequences of SARS-CoV-2, we prepared libraries using Illumina COVIDSeq RUO kits (Illumina, CA, USA) according to the manufacturer’s instructions. Sequencing was performed on a MiSeq instrument with 2 × 150-bp using a MiSeq reagent kit v2, or a NextSeq instrument using NextSeq 1000/2000 P2 Reagents (200 cycles) v3 (Illumina, CA, USA). The sequencing reads were aligned to the SARS-CoV-2 reference sequence (NC_045512.2) using BWA-MEM (Li and Durbin, 2009). We generated BAM files using SAMtools open-source software and the variants were called using AndersenLab Freyja (Karthikeyan et al., 2022). Consensus sequences were generated using the CLC Genomics workbench version 24.0.2 (QIAGEN Digital Insights, Aarhus, Denmark). The Phylogenetic Assignment of Named Global Outbreak Lineages PANGOLIN) software tool^1^ was employed for lineage calling.

### 2.4 Whole-genome sequencing of the clinical specimen collection

Nasopharyngeal and oropharyngeal swabs were collected from patients infected with SARS-CoV-2 and confirmed by real-time reverse transcription-PCR (RT-PCR; cycle threshold (Ct) < 30 for the E and N genes). Total RNA was extracted using a MagNA Pure 96 system (Roche, Basel, Switzerland) according to the manufacturer’s instructions. All clinical specimens were handled in a biosafety cabinet according to the laboratory biosafety guidelines of the KDCA. Library preparation and sequencing were conducted as described for wastewater samples, using the Illumina COVIDSeq RUO kits and MiSeq or NextSeq instruments. Total reads were trimmed, and low-quality reads were filtered using the CLC genomics workbench version 24.0.2 (QIAGEN Digital Insights, Aarhus, Denmark). The filtered reads were mapped to the reference sequence of SARS-CoV-2 (NC_045512.2) and consensus sequences were extracted. The SARS-CoV-2 lineages were analyzed using the PANGOLIN software tool (see text footnote 1). A data quality check was conducted using the Nextclade (Ns missing < 3000)^2^. Genomic sequences were deposited in the GISAID database^3^. We used only filtered complete sequences (identified as genomes of >29,000 nucleotides) in the analysis.

### 2.5 Viral load calculator

The Ct value of the ORF1ab gene analyzed by TAC was determined by converting the viral concentration into copies/mL using the ct2vl program (Hill et al., 2024).

### 2.6 Statistical analysis

All statistical analyses were performed using Microsoft Excel (Microsoft Corp., WA, USA). We conducted an *F*-test to compare variances between the coverages of MiSeq and NextSeq, followed by a two-sample *t*-test to assess the differences in group means. A chi-square test was conducted to analyze the distribution of the number of individuals by each system in wastewater and clinical samples. We used Spearman’s rho correlation analysis for analysis of the number of confirmed cases and virus concentration. The statistical significance was set at *p* < 0.05.

## 3 Results

### 3.1 SARS-CoV-2 gene concentration in wastewater by WWTPs

**FIGURE 1:**
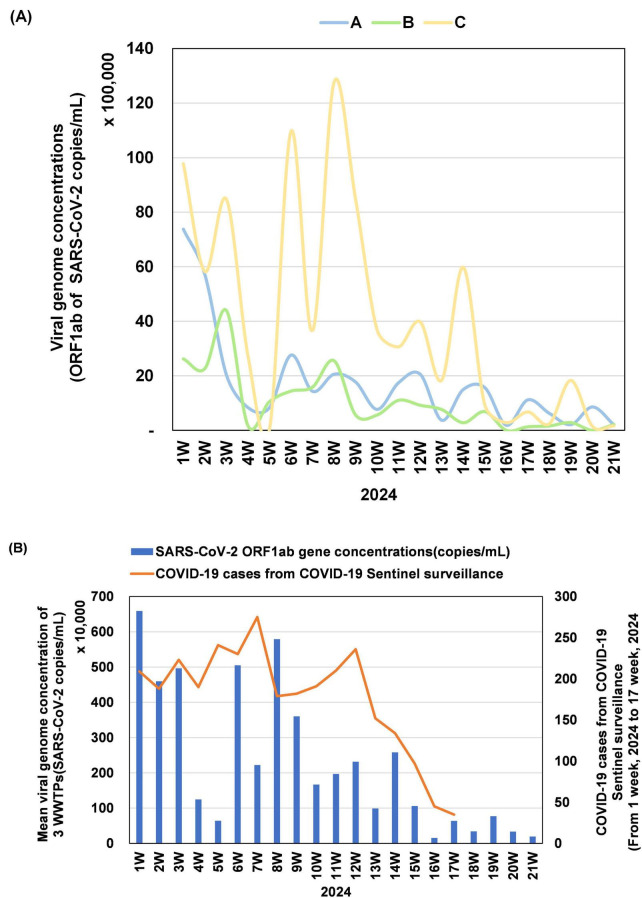
Temporal variation in SARS-CoV-2 concentrations in wastewater samples from three sites (A, B, and C) between epidemiological weeks 1 and 21 of 2024. **(A)** Quantification of SARS-CoV-2 RNA targeting the ORF1ab gene is presented in copies/mL and scaled in units of 100,000. For WWTP B, weeks 16 and 20 showed no detectable signals for ORF1ab and were recorded as zero. **(B)** The bar graph shows the mean SARS-CoV-2 ORF1ab gene concentration (copies/mL) across the three WWTPs, scaled in units of 10,000. The line graph represents weekly confirmed COVID-19 cases identified through COVID-19 sentinel surveillance in Gwangju, with values presented up to week 17, after which the surveillance program concluded.

The SARS-CoV-2 RNA concentrations (ORF1ab gene, copies/mL) were quantified weekly in influent samples from three WWTPs (A, B, and C) in Gwangju (Figure 1A). At week 1, WWTP C exhibited the highest viral concentrations at 9,779,867 copies/mL, reaching a peak of 12,762,680 copies/mL through three distinct surges at week 8. Although the levels subsequently decreased, a transient increase was observed at week 14, followed by consistently low concentrations up to week 21. In contrast, WWTP A recorded an initial concentration of 7,375,813 copies/mL, followed by a continuous decline with minor week-to-week fluctuations, ultimately stabilizing at low levels. At week 1, WWTP B recorded 2,617,887 copies/mL, followed by a temporary rise to 4,388,784 copies/mL at week 3, and a sharp decline at week 4. From week 16 onward, the viral concentrations at WWTP B remained near or below the detection limit. Overall, beginning at approximately week 10, all three WWTPs exhibited a downward trend in SARS-CoV-2 concentrations.

We further analyzed the correlation between the average SARS-CoV-2 concentration across the three WWTPs and the number of confirmed COVID-19 cases reported through COVID-19 sentinel surveillance in Gwangju (Figure 1B). The average viral concentration peaked at week 1 at approximately 6,591,189 copies/mL, decreased to 641,331 copies/mL at week 5, and increased again at week 8 to 5,789,987 copies/mL. A gradual decline followed, with concentrations dropping below 100,000 copies/mL after week 16. COVID-19 sentinel surveillance recorded 209 confirmed cases at week 1, with a steady increase to a peak of 275 cases at week 7, followed by a marked decline after week 13. The trends in average SARS-CoV-2 concentrations and confirmed case counts displayed concurrent increases at approximately weeks 7–8 and synchronous decreases after week 14, suggesting an overall alignment of temporal dynamics during the study period (Spearman’s correlation ρ = 0.343, *p* = 0.178).

### 3.2 Comparison of SARS-CoV-2 sequencing data between MiSeq and NextSeq using wastewater samples

To obtain full-length SARS-CoV-2 genome sequences from the collected wastewater samples, next-generation sequencing (NGS) was performed using the Illumina MiSeq and NextSeq platforms. The MiSeq platform generated an average of 858,019 total reads, with a mean sequencing depth of 3,556×, whereas the NextSeq platform yielded 18,853,854 reads with an average depth of 63,403× (Supplementary Table 1).

**FIGURE 2:**
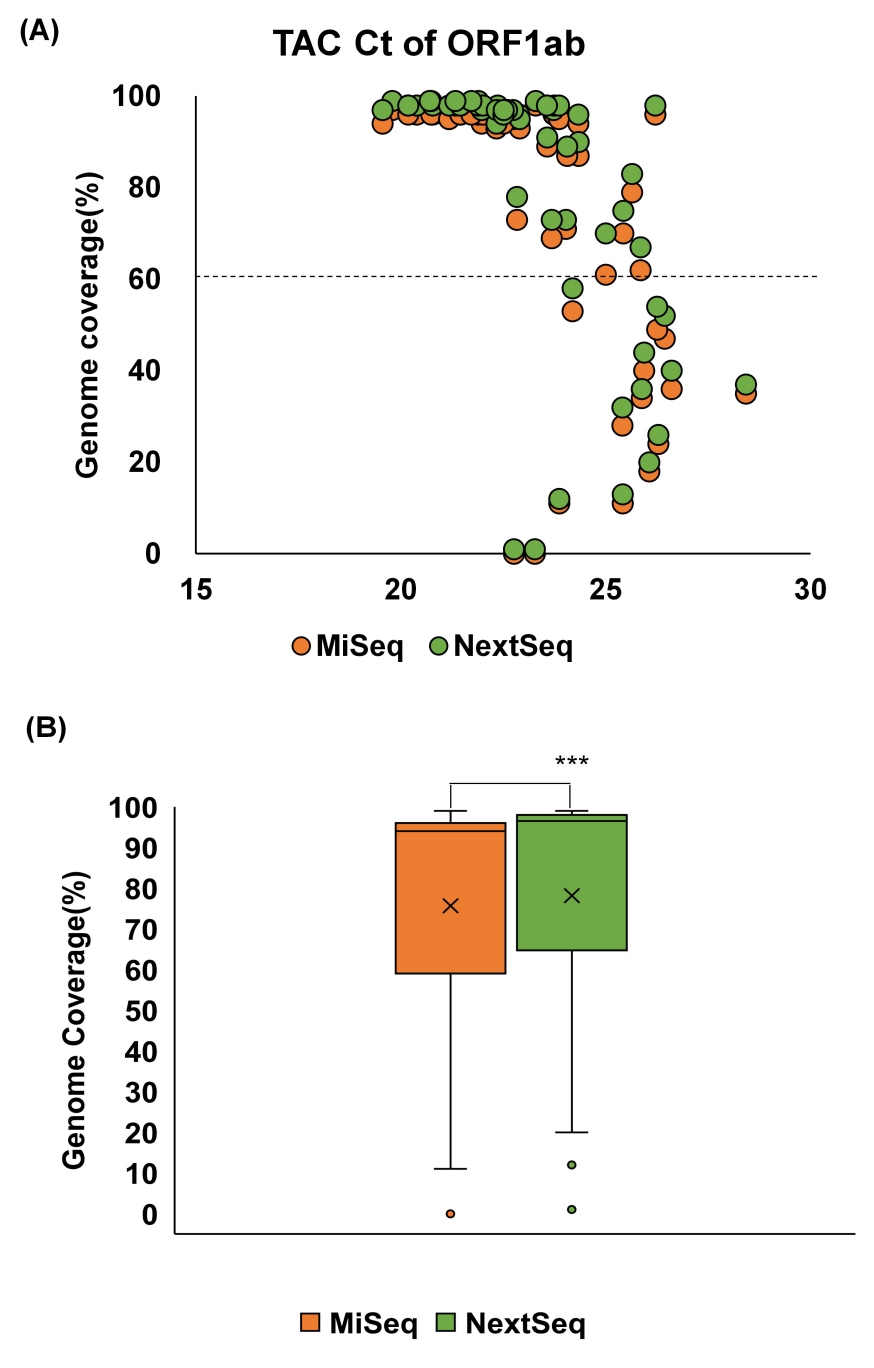
Relationship between Ct values of the SARS-CoV-2 ORF1ab gene and genome coverage (%) by sequencing platform. Genome coverage (%) derived from WGS is plotted against the Ct values of each sample, with the results shown separately for the MiSeq (orange) and NextSeq (green) platforms. Dashed line indicates the threshold for 50% genome coverage. **(A)** Among 70 wastewater samples collected between January and May 2024, 58 samples with available TAC Ct values for the ORF1ab gene were included in the analysis. **(B)** Comparison of genome coverage between the MiSeq and NextSeq platforms. Statistical significance was assessed using a *t*-test (***p < 0.001).

The correlation between the genome coverage and Ct values was evaluated using TAC assays. In both platforms, lower Ct values were associated with higher SARS-CoV-2 genome coverage (Figure 2A). Specifically, samples with Ct values below 22 achieved >90% genome coverage across both platforms, whereas the genome coverage declined sharply with increasing Ct values. In particular, samples with Ct values ≥ 25 rarely exceeded 50% genome coverage. Comparative analysis of genome coverage across all samples showed that the NextSeq platform provided a significantly higher mean genome coverage (76.5%) than MiSeq (72.6%) (Figure 2B).

### 3.3 Comparison of distribution of SARS-CoV-2 sublineages in clinical and wastewater samples

**FIGURE 3:**
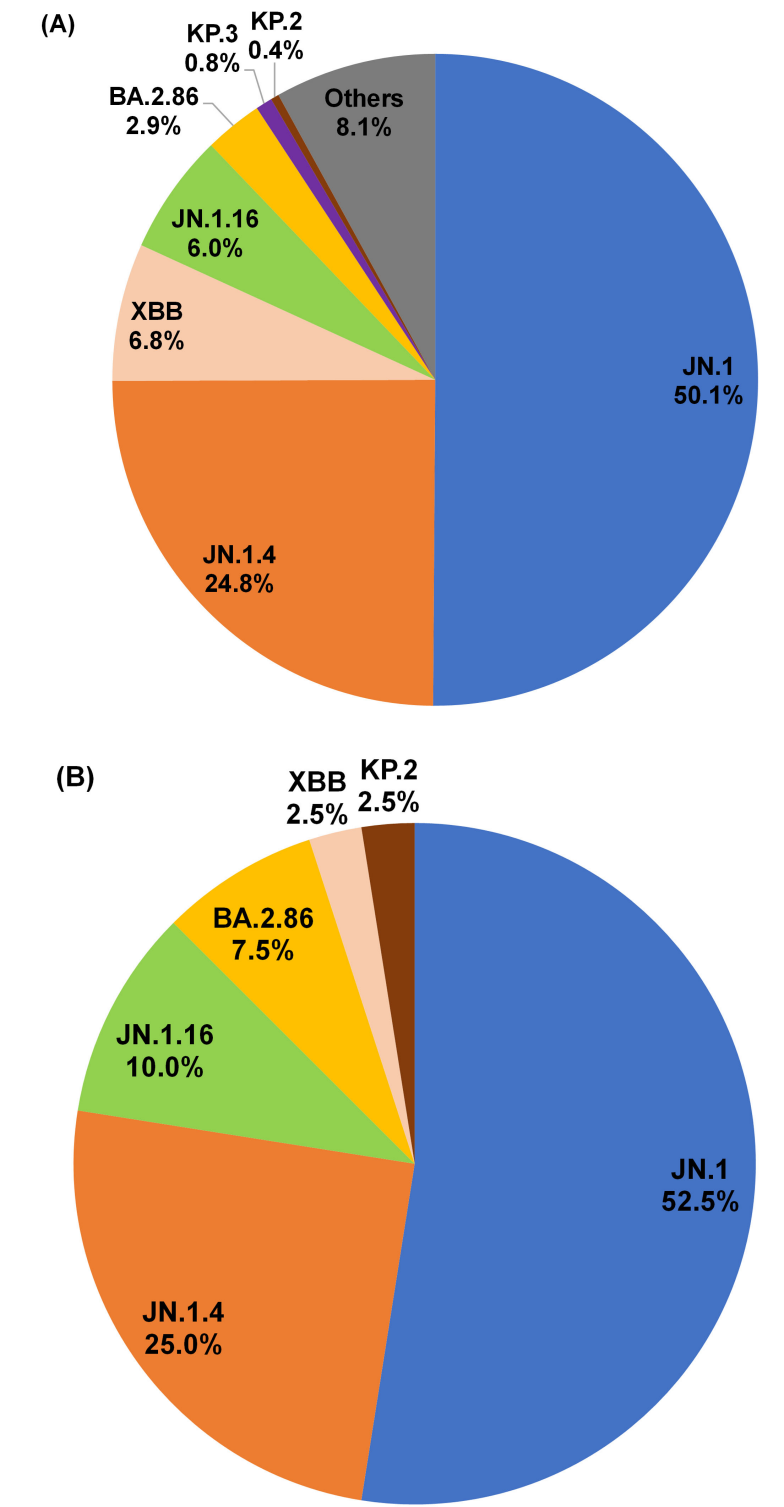
Lineage distribution of SARS-COV-2 in clinical specimen and wastewater samples. Lineage proportions are shown as percentages and, unless indicated otherwise, they include sublineages within each major lineage. The “JN.1” category includes all JN.1-derived sublineages except JN.1.4, JN.1.16, KP.3, and KP.2. **(A)** Lineage analysis of 483 clinical specimens collected through COVID-19 sentinel surveillance in Gwangju between weeks 1 and 17 of 2024. **(B)** Lineage analysis of 40 wastewater samples with ≥50% genome coverage, out of a total of 58 collected.

The NGS data obtained from the wastewater samples were analyzed using the AndersenLab Freyja pipeline to identify the dominant SARS-CoV-2 sublineages. Subsequently, these sublineages were compared with those detected in contemporaneous clinical specimens collected through COVID-19 sentinel surveillance in Gwangju (Supplementary Table 2). In the clinical samples, JN.1 was the most prevalent lineage, accounting for 50.1% (*n* = 242) of all detected sublineages, followed by JN.1.4 (24.8%, *n* = 120), XBB sublineages (6.8%, *n* = 33), JN.1.16 (6.0%, *n* = 29), BA.2.86 (2.9%, *n* = 14), KP.3 (0.8%, *n* = 4), KP.2 (0.4%, *n* = 2), and other sublineages (8.1%, *n* = 39) (Figure 3A). Among wastewater-derived sequences, JN.1 sublineages were dominant at 52.5% (*n* = 21) of detections, followed by JN.1.4 (25.0%, *n* = 10), JN.1.16 (10.0%, *n* = 4), BA.2.86 (7.5%, *n* = 3), and both XBB and KP.2 at 2.5% (*n* = 1) each. Notably, the proportions of JN.1 and JN.1.4 were comparable between the clinical and wastewater samples at 50.1% vs. 52.5% and 24.8% vs. 25.0%, respectively (χ^2^(7, *N* = 16) = 10.81, *p* = 0.147) (Figure 3B).

## 4 Discussion

As wastewater-based surveillance captures pathogens excreted by all infected individuals, including symptomatic, asymptomatic, and undiagnosed cases, it can serve as a proxy for community-level transmission intensity during infectious disease outbreaks (Starke et al., 2024). Accordingly, countries such as the United States and Canada have implemented national wastewater-based surveillance systems to supplement SARS-CoV-2 monitoring using conventional clinical approaches.

The current study was conducted as part of a wastewater-based infectious disease surveillance project led by the KDCA, a national central government institution. This study is the first to directly compare the correlation between SARS-CoV-2 WWTP samples and clinical specimens in Gwangju, as well as perform whole-genome sequencing on both. This approach facilitated more comprehensive and precise surveillance of the SARS-CoV-2 variants circulating in the local community (Kim et al., 2023, 2024). The results show that the average SARS-CoV-2 virus concentration at three wastewater treatment plants in the Gwangju area and the number of COVID-19 confirmed cases identified through sample surveillance in the same area generally showed similar increasing and decreasing trends (Spearman rho = 0.343, *p* = 0.178). This finding suggests that the SARS-CoV-2 virus concentration measured through wastewater surveillance could serve as an indirect indicator of the infection trends within a community. However, according to a literature review by Bertels et al. (2022), wastewater-based SARS-CoV-2 genetic analysis could be influenced by various external factors starting from the pre-sampling stage. Such factors include the viral shedding characteristics of infected individuals (timing and amount of shedding), population size, physical and chemical conditions within the sewage system (temperature, pH, solids content, flow rate, retention time), and the sampling strategy. Such variability could potentially cause a low direct correlation between viral concentrations in wastewater and the number of confirmed clinical cases (Li et al., 2021; Hassard et al., 2022).

Interestingly, during the overall sampling period, WWTP C, despite serving a smaller population, consistently exhibited higher mean SARS-CoV-2 concentrations compared with those of WWTP A and B, which treated wastewater from larger populations. This discrepancy could be ascribed to WWTP A and B also processing substantial volumes of industrial effluent from large manufacturing facilities. Similarly, previous studies have reported that WWTPs co-treating industrial wastewater tended to show lower SARS-CoV-2 concentrations than plants primarily processing domestic wastewater (Al-Duroobi et al., 2023). These findings suggest that environmental factors, including the composition of influent wastewater, can influence the detectability and concentration of viral pathogens during wastewater-based surveillance.

Segelhurst et al. (2023) reported that securing a sequencing depth greater than 10 × facilitates reliable coverage of over 90% of the SARS-CoV-2 genome in wastewater samples. However, Lipponen et al. (2024) observed that uneven sequencing depths in wastewater-derived genomes can reduce the coverage of certain genomic regions, potentially limiting the sensitivity for variant detection.

In this study, sequencing data generated from wastewater using the Illumina MiSeq and NextSeq platforms were analyzed comparatively with respect to data quality metrics, including total read count, sequencing depth, and genome coverage. The NextSeq platform demonstrated superior performance across all metrics, suggesting enhanced sensitivity for detecting low-abundance pathogens (*p* < 0.001). However, standardized guidelines for optimal sequencing depth and genome coverage thresholds in wastewater samples are yet to be established. This deficiency highlights the need for clear recommendations regarding platform selection and sequencing strategies for future large-scale wastewater surveillance programs.

Further, we compared the distribution of SARS-CoV-2 sublineages in the wastewater and clinical samples concurrently collected in Gwangju. During the study period, which coincided with a rise in JN.1 sublineages and decline in XBB variants, similar proportions of JN.1-derived lineages were observed in both surveillance streams. These findings support the feasibility of using wastewater-based surveillance as a complementary tool to clinical sequencing for monitoring variant dynamics in communities. Previous studies have demonstrated that novel variants are detected occasionally in wastewater prior to their identification in clinical specimens. This finding underscores the potential of wastewater-based surveillance for the early warning of emerging variants (Jahn et al., 2022; Wilhelm et al., 2022).

This study has several limitations that should be considered when interpreting the results. First, not all SARS-CoV-2 positive wastewater and clinical samples collected in the Gwangju area were included in the genomic analysis, and genomes with low coverage were excluded from the analysis. Therefore, the limited sample size probably does not fully reflect the genomic diversity of SARS-CoV-2 in wastewater, and rare or low-frequency variants may have been missed, potentially leading to an underestimation of population diversity. Additionally, although samples were collected every Monday at the same time to minimize variation in sampling time, variables such as environmental factors and demographic characteristics were not considered, which could affect the interpretation of the results. These limitations could impact the statistical reliability and reproducibility of the study findings, and cautious interpretation is required when generalizing these results to other regions or time periods. Therefore, in future studies, sample size must be increased, analytical methods developed that include genomes with low coverage, and various factors considered to obtain more comprehensive and generalizable data.

## 5 Conclusion

This study evaluated the feasibility of wastewater-based SARS-CoV-2 surveillance in Gwangju by analyzing viral concentrations and variant distributions in wastewater and comparing them with contemporary clinical surveillance data. The results demonstrate that fluctuations in wastewater SARS-CoV-2 concentrations closely mirrored the trends in the reported cases, suggesting that wastewater-based surveillance could be a reliable indicator of community transmission dynamics. Moreover, the distribution of viral variants observed in the wastewater samples was consistent with that in the clinical specimens, supporting the utility of wastewater-based sequencing for monitoring variant circulation. These findings highlight the potential of wastewater surveillance as an effective public health tool to complement clinical surveillance, thereby enabling the early detection and monitoring of emerging variants at the community level. The ongoing expansion and standardization of wastewater-based surveillance systems are expected to enhance infectious disease preparedness and response capacity.

## Data Availability

The original contributions presented in the study are publicly available. This data can be found here: http://www.ncbi.nlm.nih.gov/bioproject/1332254.

## Footnotes

1
https://github.com/cov-lineages/pangolin

2
https://clades.nextstrain.org

3
https://www.gisaid.org/
